# Experimental Investigation of the Effect of Solvent Type and Its Concentration on the Performance of ES-SAGD

**DOI:** 10.3390/mps8020039

**Published:** 2025-04-08

**Authors:** Sajjad Esmaeili, Brij Maini, Zain Ul Abidin, Apostolos Kantzas

**Affiliations:** 1 Department of Chemical and Petroleum Engineering, University of Calgary, Calgary, AB T2N 1N4, Canada; bmaini@ucalgary.ca (B.M.); zain.ulabidin1@ucalgary.ca (Z.U.A.); akantzas@ucalgary.ca (A.K.); 2 Computer Modelling Group (CMG), Calgary, AB T2L 2M1, Canada

**Keywords:** bitumen, thermal recovery, ES-SAGD, effect of solvent composition

## Abstract

Steam-assisted gravity drainage (SAGD) is a widely used thermal enhanced oil recovery (EOR) technique in North America, particularly in high-permeability oil sand reservoirs. While effective, its economic viability has declined due to low oil prices and high greenhouse gas (GHG) emissions from the steam generation. To improve cost-effectiveness and reduce emissions, solvent-assisted SAGD techniques have been explored. Expanding Solvent-SAGD (ES-SAGD) involves co-injecting light hydrocarbons like propane or butane with steam to enhance oil viscosity reduction. This approach lowers the steam–oil ratio by combining solvent dissolution effects with thermal effects. However, the high cost of solvents, particularly butane, challenges its commercial feasibility. Propane is cheaper but less effective, while butane improves performance but remains expensive. This research aims to optimize ES-SAGD by using a propane–butane mixture to achieve efficient performance at a lower cost than pure butane. A linear sand pack is used to evaluate different propane/butane compositions, maintaining constant operational conditions and a solvent concentration of 15 vol.%. Temperature monitoring provides insights into steam chamber growth. Results show that solvent injection significantly enhances ES-SAGD performance compared to conventional SAGD. Performance improves with increasing butane concentration, up to 80% butane in the C_3_–C_4_ mixture at the test pressure and ambient temperature. Propane alone results in the lowest system temperature, while conventional SAGD reaches the highest temperature. These findings highlight the potential of optimized solvent mixtures to improve ES-SAGD efficiency while reducing costs and GHG emissions.

## 1. Introduction

Thermal enhanced oil recovery (TEOR) includes several techniques to recover heavy oil and bitumen from buried oil formations, in which heat plays a paramount role in reducing the viscosity of bitumen. These include cyclic steam stimulation (CSS), steam-assisted gravity drainage (SAGD), in situ combustion (or air injection), and steam flooding. SAGD is the most widely applied TEOR technique for the recovery of bitumen or highly viscous heavy oil in Canada [1]. Generally, two horizontal wells are used in an SAGD well configuration, where there is about a five-meter gap between the top well (injector) and bottom well (producer) [2,3]. The vertical permeability of the formation and viscosity of bitumen have a significant impact on the steam chamber expansion rate, which controls the oil production rate in this technique [4,5,6,7]. Although oil production with this technique has been commercially viable, the low oil prices in recent years have made this technique less cost-effective, especially in reservoirs that do not have the ideal characteristics for its applicability. Moreover, SAGD requires burning large quantities of fossil fuels for steam generation, which results in the release of large quantities of greenhouse gases. Therefore, the development of new thermally enhanced oil recovery techniques that are more cost-effective and environmentally responsible has become a priority.

Adding a small amount of hydrocarbon solvent, either heavy or light, to the injected steam in SAGD, known as Expanding-Solvent SAGD (ES-SAGD), is a promising TEOR approach that brings some advantages from VAPEX to SAGD [8]. Fundamentally, the idea behind the co-injection of the solvent with steam is to enhance the viscosity reduction, besides other possible effects such as relative permeability modification, fluid/fluid and rock/fluid interaction variations, and the reduction of heat loss to the overburden rock formation. The ability of the solvent to mix with the bitumen depends on its solubility, polarity, and molecular size, which influence the degree of viscosity reduction. Additionally, steam helps to increase the temperature, facilitating the solvent’s penetration into the bitumen and promoting a better solvent–bitumen interaction. This interaction not only reduces viscosity but also lowers the interfacial tension between the solvent and bitumen, improving the solvent’s efficiency in displacing bitumen. The combined effects of solvent, steam, and heat result in the enhanced mobility of the bitumen, which is crucial for the success of ES-SAGD. These chemical interactions are key to optimizing the overall performance of the process and enhancing oil recovery. The promising outcomes from the application of ES-SAGD have been confirmed in field-scale [9,10] and in several lab-scale experimental studies [11,12,13,14]. As the interactions between fluid/fluid and rock/fluid strongly depend on the type of oil and rock formation and other effective parameters that vary from reservoir to reservoir, extensive laboratory and field-scale studies need to be carried out before its commercial application [15].

By looking into the literature, it can be found that different types of solvents or gases were employed in several studies to enhance the efficiency of SAGD and reduce the water requirement. The gaseous solvents involved in these studies were carbon dioxide [16], nitrogen [17], and propane [18,19], while the liquid solvents included n-pentane [20], n-hexane [21], etc. [22,23,24]. Several reported studies selected propane or butane co-injected with steam [18,22,23,25,26].

Yongbin et al. [5] conducted one SAGD and two ES-SAGD experiments in a physical model using two types of solvent, including 10% n-hexane, and 9% n-hexane + 1% xylene. They added xylene to the system because it has a perfect asphaltene dissolution ability, which can eliminate formation plugging. The results confirmed that the shape of the steam chamber and its expansion rate were different for these cases. In addition, the lower temperature of the steam chamber (10–20 °C lower than the SAGD experiment) was achieved in the solvent-added cases. The lateral steam chamber expansion was higher in the case of ES-SAGD, where added xylene even improved the oil production rate by reducing the flow resistance [5]. In another study, Ardali et al. [25] conducted a simulation study and asserted that butane is the best option to be injected with steam to recover the bitumen from the cold lake reservoir, in comparison with other solvents. The core flooding experiments accomplished by Coelho et al. [18] in 2017 have proven that the co-injection of propane with steam can increase the ultimate oil recovery in these types of experiments. The bitumen involved in this study came from Peace River. In contrast to the previous study, Li et al. [22,23] concluded that the injection of propane with steam leads to a reduction in oil production rate. According to the study conducted by Dong [27], the concentration of solvent in the steam chamber is lower for heavier solvents compared to lighter solvents. As a result, a higher temperature can be achieved in this region. In addition to these studies, Eghbali et al. [28] also mentioned some reasons for the observed difference between the performance of the ES-SAGD using propane and ES-SAGD using butane. The difference was mostly due to the viscosity of the bitumen at the edge of the steam chamber. Since the solubility of propane is lower than butane in the bitumen and the temperature of the steam chamber in the propane case is lower than the butane case, the lower viscosity of bitumen at the edge of the chamber was captured for the butane case. In a recent simulation study [28], an optimum composition for the injection of pure propane or pure butane with steam was obtained. Since butane is considerably more expensive than propane, the optimization of the butane concentration in mixtures of propane and butane injected with steam becomes an economically important consideration.

## 2. Motivation

The objective of this study was to experimentally determine whether a mixture of propane and butane performs as well as, or better than, pure butane when used as a solvent in the ES-SAGD process under identical conditions. Although previous studies have examined ES-SAGD performance at both field and laboratory scales, there are still many uncertainties about how to optimize this technique. Adjusting the operational conditions, amount of solvent added, or solvent composition could help to improve efficiency. Recently, propane and butane have gained attention as cost-effective solvent options for ES-SAGD. However, no experimental studies have identified the optimal butane/propane mixture that maximizes oil recovery while minimizing the steam-to-oil ratio. This research aims to fill that gap by conducting multiple SAGD and ES-SAGD experiments in a linear sand pack using Athabasca bitumen, butane, propane, and their mixtures. Four different butane/propane compositions were tested to optimize the injected solvent mixture. During the experiments, temperatures in different parts of the steam chamber were monitored to track chamber expansion and heat distribution. Additionally, oil recovery, oil production rate, and the steam-to-oil ratio were evaluated to assess overall performance. Solvent recovery was not within the scope of this research, as a larger-scale system is required for a more accurate assessment.

## 3. Experimental Design

### 3.1. Core Flooding Setup Description

**Figure 1:**
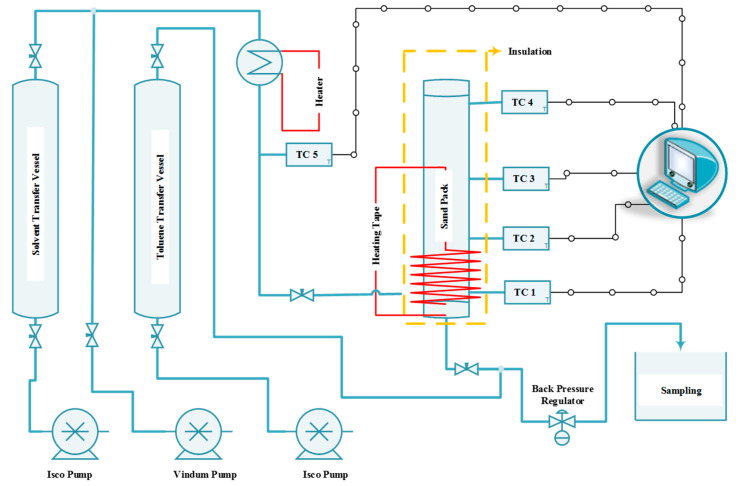
Schematic of the experimental rig used to run SAGD and ES-SAGD experiments.

A simple setup was designed and constructed for this research and was capable of co-injecting steam and solvent into the sand pack, monitoring and recording the temperature at various locations in the system, and measuring the oil production rate. Figure 1 shows the schematic of the experimental rig developed in this study.

As shown in this figure, the system includes three different positive displacement pumps (two ISCO 500D pumps for the solvent and toluene, and one Vindum pump for water) to inject the solvent solution, water, and toluene into the system. It also features a back pressure regulator (BPR) to maintain the outlet pressure at the desired level, two transfer vessels—one for toluene and the other for the solvent solution—an in-line steam generator to convert the injected fluids (including water and solvent) into vapor, several thermocouples to monitor temperature across the sand pack, a data acquisition system to record pressure and temperature, several heating tapes, an insulation blanket, and a sand holder. The pumps can continuously inject fluid with an accuracy of 0.0001 cm^3^/min. The pressure on the BPR was set at 200 psi to ensure that the water would be in the vapor phase when the temperature exceeded 205 °C. The toluene transfer vessel was included to facilitate the BPR’s function, especially during the early stage of production when the proportion of bitumen in the produced fluid is high and may interfere with smooth BPR operation. The sticky nature of bitumen can adversely affect the BPR, and the toluene helps dilute the bitumen, eliminating its sticky properties. This solution for eliminating BPR problems was proposed and successfully tested by Esmaeili et al. [29,30], where it effectively tackled the issue. The toluene injection port was located downstream of the sand pack and upstream of the BPR (see Figure 1) to minimize its impact on test performance.

**Figure 2:**
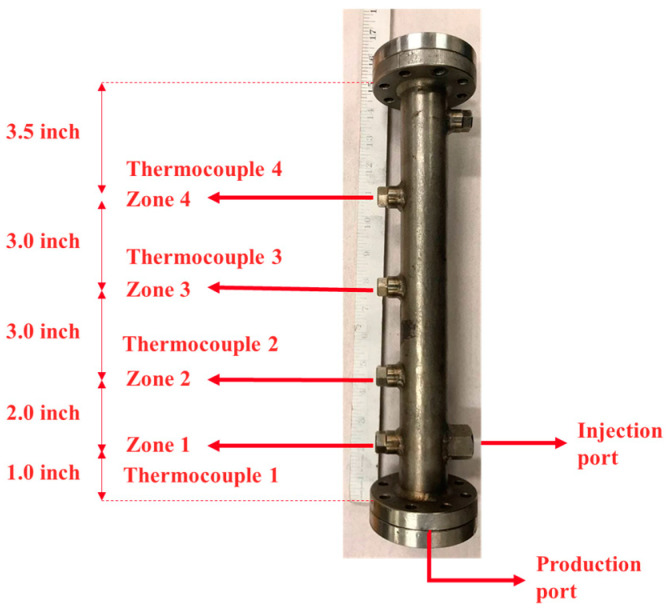
The sand holder used in this study with four ports for thermocouples.

Heating tape was installed on the sand pack between the injection port and the production port. The purpose of the heating tape was to preheat a small portion of the sand pack and generate suitable fluid conductivity between the injection and production ports by reducing bitumen viscosity before steam injection. The sand pack was equipped with four thermocouples, located at distances of 2.54 cm, 7.62 cm, 15.24 cm, and 22.86 cm from the production port. The distance between the injection port and the production port was approximately 2.54 cm, with the injection port positioned above the production port to mimic the well configuration in the SAGD process. Figure 2 shows the sand holder used in this study, along with its dimensions.

### 3.2. Materials

#### 3.2.1. Sand Pack Properties

The porous media consisted of clean silica sand particles with a mesh size of 12–20. The sand particles were confined within a cylindrical space. The sand pack had a maximum length of 31.60 cm and an inner diameter of 2.54 cm, resulting in a cross-sectional area of 5.07 cm^2^ and a total volume of 160.12 cm^3^. One end cap of the sand holder was fixed, while the height of the other end cap was adjustable through the axial movement of a 2.5 cm long cylindrical stem containing sealing O-rings. As a result, the total length of the porous media varied between 30.88 cm and 31.60 cm across different experiments. This movable stem allowed the application of axial stress on the sand by tightening the end cap bolts. The same torque was applied to the bolts in all tests to maintain consistent axial stress on the sand. All experiments were conducted with freshly packed sand. The porosity and permeability of the sand packs were approximately 36.40% ± 0.46% and 358.41 ± 66.7 Darcy, respectively.

#### 3.2.2. Fluid Properties

The oil phase was Athabasca bitumen with a density of 1.026 g/cm^3^ (at 23 °C and 400 psi). The density and viscosity of employed bitumen at different temperatures and a fixed pressure were measured earlier and reported in our previous research [29,30,31,32,33]. In addition, the SARA test analysis of this bitumen has been reported elsewhere [29]. The measured density of the bitumen at different temperatures showed a linear trend. Equation (1) expresses the corresponding correlation. The bitumen viscosity at the ambient pressure at several temperatures and the water viscosity at 380 psi and several temperatures are provided in a study conducted by Esmaeili et al. [29]. Equation (3) expresses the best-fitted correlation of bitumen viscosity as a function of temperature.

The aqueous phase was deionized water in all experiments. The density of deionized water was also measured using our developed HP-HT Pycnometer, and the results can be found in our previous research [29]. Additionally, Equation (2) expresses the best-fitted correlation on the measured data points of the aqueous phase. Likhachev [34] developed a non-linear empirical correlation for the pure water viscosity as a function of pressure and temperature, as expressed in Equation (4). These equations are expressed as follows:

(1) $$\rho_{Bit}=1.0414+6.97\times 10^{-3}T$$

(2) $$\rho_{Wat}=-2\times 10^{-6}T^{2}-3\times 10^{-4}T+1.0089$$

(3) $$\mu_{Bit}=\exp\left(\exp\left(7.776\left(\ln\left(T\right)\right)-1.721{\left(\ln\left(T\right)\right)}^{2}+0.112{\left(\ln\left(T\right)\right)}^{3}-8.498\right)\right)$$

(4) $$\mu_{w}=2.4055\times 10^{-2}\exp\left(4.42\times 10^{-4}P_{w}+\frac{4.753-90.565P_{w}}{8.314\times 10^{-3}\left((T+273.15)-139.7-1.24\times 10^{-2}P_{w}\right)}\right)$$

where *T* is in °C, $\rho_{wat}$ is in g/cm^3^, $\rho_{bit}$ is in g/cm^3^, and $P_{w}$ is in bar.

Two different hydrocarbon solvents, propane (C_3_) and butane (C_4_), were used in this study. Since both propane and butane are gaseous at ambient temperature and pressure, two 250 cm^3^ transfer vessels were employed to prepare their mixtures. The first transfer vessel was used to extract gas or a gas/liquid mixture from the gas tank. The vessel was then pressurized to over 200 psi at ambient temperature to convert the gas phase into the liquid phase. As a result, only the liquid phase of propane or butane at ambient temperature and 200 psi remained in the vessel. The liquefied gas was then transferred to the second transfer vessel, where a metered volume of the liquid phase was maintained under pressure for the experiment. Thus, the first transfer vessel liquefied the extracted gas, while the second transfer vessel collected the liquefied gas for testing or mixing purposes. To prepare a specific propane/butane mixture, the same procedure was repeated twice, transferring metered volumes of each component into the second transfer vessel. This process allowed the desired mixture to be achieved.

### 3.3. Experimental Procedure

This section describes the experimental procedure used to conduct the SAGD or ES-SAGD experiments in the present study [29], as follows:

- The clean and dry sand pack is first weighed using an accurate balance, then flushed with carbon dioxide gas for 20 min to displace the trapped air. Afterward, it is evacuated to remove the carbon dioxide gas from the system. Once the evacuation is complete, the sand pack is saturated with deionized water. At the end of this stage, the weight of the sand pack is measured again. The difference between the weight of the dry sand pack and the wet sand pack represents the weight of the imbibed water in the sand pack.
- The sand pack is placed in an oven at 75 °C for a couple of hours to preheat the system before oil flooding. Similarly, the oil transfer vessel is heated to the same temperature to mobilize the bitumen. The bitumen is then injected into the sand pack at 75 °C with a flow rate of 1 cm^3^/min until oil breakthrough occurs. Oil flooding is terminated after the breakthrough, and the mass of produced water is measured to determine the irreducible water saturation remaining in the system at the end of this stage.
- Before starting steam (or steam/solvent) injection, the power to the heating tape installed between the injection and production ports on the sand pack is switched on. The system is heated until the temperature in zone (I) reaches 80 °C. This preheating stage is kept the same for all tests. Meanwhile, steam or steam/solvent injection begins in the steam generator, with the set temperature maintained at 235 °C using a temperature controller. This temperature is consistent across all tests. However, the fluid initially flows through the bypass line and is produced from the BPR without contacting the sand pack. This step continues for over an hour to ensure that both the production and injection lines are sufficiently heated before bitumen production begins.
- Once the system is sufficiently heated and the temperature of thermocouple #1 reaches 80 °C, steam or steam/solvent is injected into the sand pack at a constant total flow rate of 2.0 cm^3^/min using a constant flow pump. The outlet pressure is set on the BPR and installed at the outlet to a fixed pressure lower than the steam pressure. This set pressure is kept the same across all core flooding tests. At this point, no additional heat is introduced into the system from the heating tape. The injection continues for over six hours, during which several samples of the produced effluent are collected from the sand pack. The temperature in different zones of the sand pack is recorded and monitored for later analysis. The outlet pressure remains constant throughout the experiment.

As heat loss and steam condensation are major concerns in SAGD-type core flooding tests, the following measures were implemented during the tests:

1. Keeping the steam generator as close to the sand holder as possible and shortening the tubing. The steam generator was kept inside a box that was fully isolated, and the tubing coming from the steam generator was also wrapped with isolation material and a heating tape to maintain the temperature.
2. Installing two thermocouples, one at the outlet of the steam generator and another one at the injection port of the sand pack, to monitor the temperature of steam coming from the steam generator and before injecting into the sand pack.
3. Preheating the bottom part of the sand pack up to 80 °C prior to the test to generate the connection between injector and producer and mobilizing the bitumen.
4. Pre-circulating the steam to stabilize the steam temperature in the injector and steam generator before running the test.
5. Wrapping the whole sand pack body with a thick layer of isolation material to reduce the heat loss from the metal body of the sand pack.

### 3.4. Effluent Sample Analysis

At the start of the experiment, hot bitumen and condensed water were produced from the BPR. Due to the configuration of the BPR used in this study, a significant shear force was applied to the produced effluent when the BPR valve was opened, resulting in the formation of a strong emulsion of bitumen and water that cannot be easily separated. In both the base-SAGD and ES-SAGD experiments, we were unable to easily separate the produced water from the oleic mixture, regardless of the presence of the solvent. Therefore, the mixture, which includes both the condensed water phase and oleic phase, was transferred to a round flask for Dean–Stark analysis. Since the amount of toluene added to the mixture for BPR operation was known, its presence did not affect the bitumen weight measurement at the end of the analysis. By conducting the Dean–Stark analysis, all the aqueous phase was extracted from the mixture, allowing us to determine the weight of water. After this, the remaining mixture (containing toluene and bitumen) at the bottom of the round flask was subjected to a Rotary Evaporation process. A consistent procedure was followed to evaporate and recover as much toluene as possible from the mixture. The procedure described here has been explained in our previous research [30,31,32], showing a near-perfect toluene recovery where the performance of this procedure was double-checked with several prepared samples. The reported material balance error was less than 1% [30,31,32]. The difference between the dry weight of the flask and the weight of the flask with the settled bitumen gives the mass of the collected bitumen. This allows for the proper evaluation of the fraction of produced water and bitumen using the solvent extraction technique. The mass of the produced water and oil can be converted to volume in the test conditions based on the density of the fluids. This procedure works effectively for both SAGD- and ES-SAGD-produced effluents.

## 4. Results and Discussion

As mentioned earlier, seven different SAGD and ES-SAGD experiments were successfully conducted in this study. Pure propane and pure butane (99.9% purity) were used individually for the ES-SAGD experiments. Additionally, four ES-SAGD experiments were carried out using four different propane/butane mixtures (20%C_3_–80%C_4_, 40%C_3_–60%C_4_, 60%C_3_–40%C_4_, and 80%C_3_–20%C_4_), prepared at 200 psi and 23 °C, to determine the optimal composition for ES-SAGD. Table 1 summarizes various properties of the experimental tests, including the composition of the hydrocarbon gas mixtures, sand pack porosities, sand pack absolute permeabilities, weights of added sand, pore volumes, and the flow rates of injected steam and solvent.

As can be seen in Table 1, the porosity of the sand pack in all experiments was approximately 36.40%. Although the variation in absolute permeability to water was greater than the variation in porosity, the average permeability was around 359.41 Darcy.

### 4.1. Temperature Profile Within the Sand Pack

The temperature profile in the SAGD or ES-SAGD process is one of the most important factors in assessing the rate of SAGD chamber expansion during steam or steam/solvent injections. The temperature at four different locations in the sand pack, as shown in Figure 2, was recorded and monitored during the experiments.

**Figure 3:**
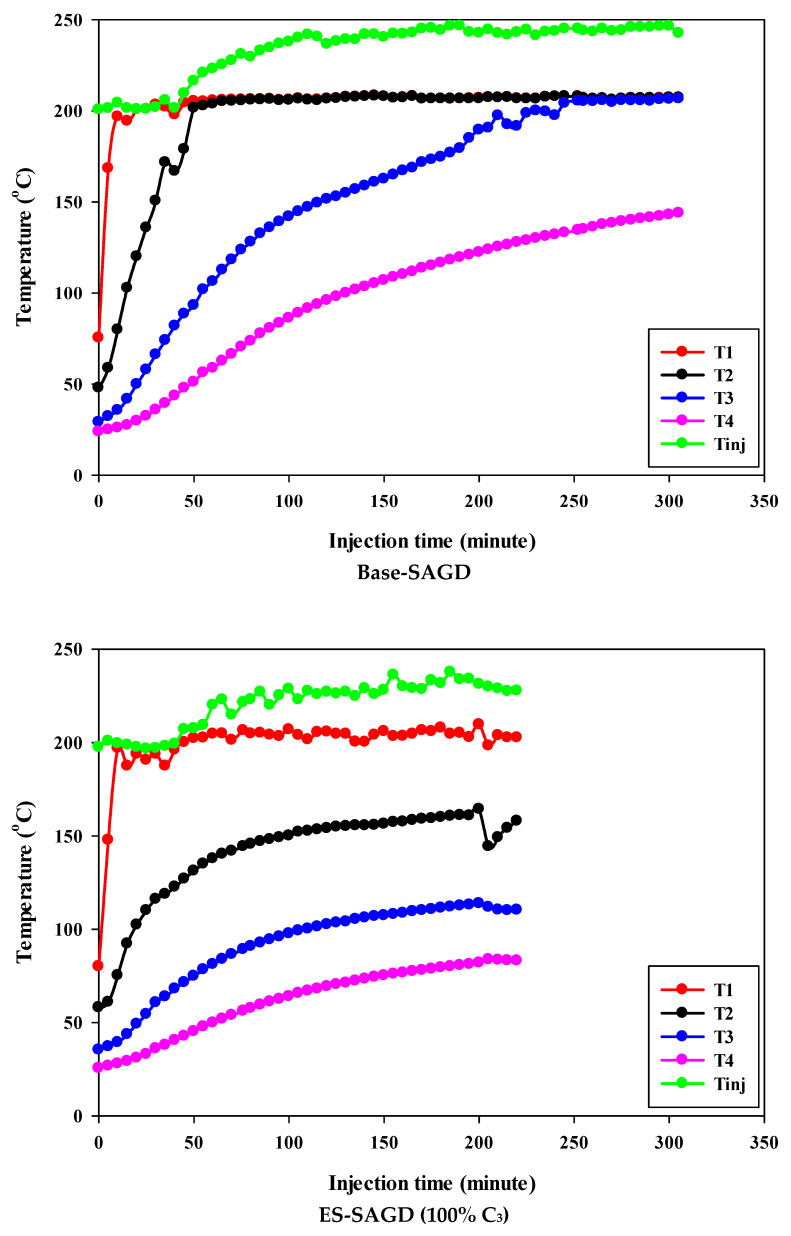

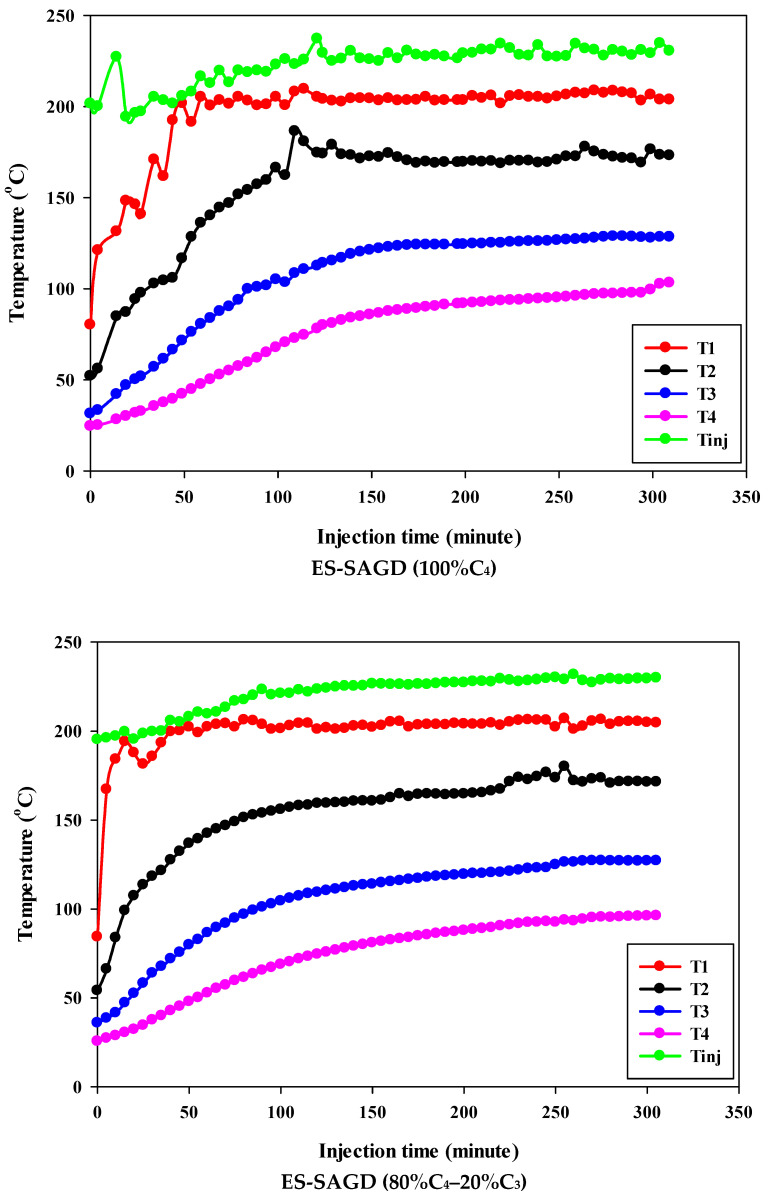

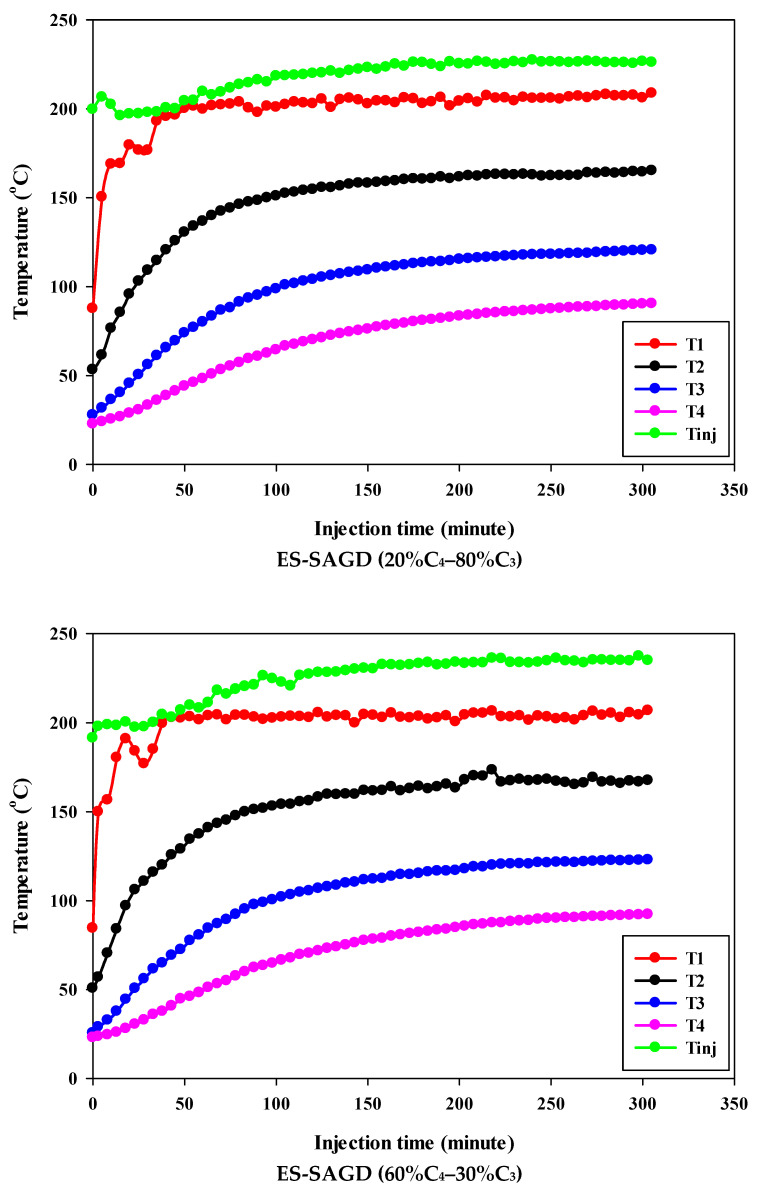

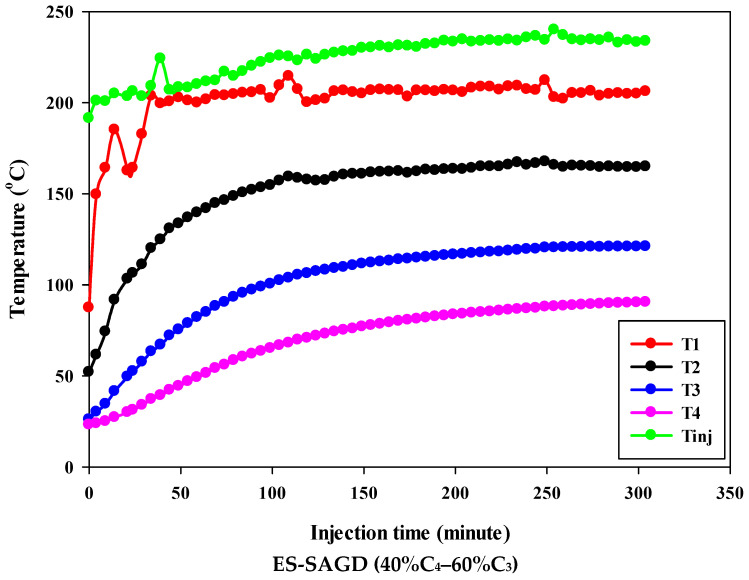
The temperature profiles within the sand pack at four different locations and the temperature of the injected fluid in different experiments.

Figure 3 shows the temperature profiles within the sand pack and the temperature of the injected fluid for different experiments. According to the experimental procedure described earlier, the experiment began once the temperature of Zone I reached 80 °C. Therefore, at the start of all experiments, the temperature of Zone I was around 80 °C. At that point, the temperatures of Zone II, Zone III, and Zone IV were approximately 50 °C, 27 °C, and 25 °C, respectively. Additionally, the temperature of the injected fluid at the start of the experiments was about 200 °C. Once steam injection began, the temperature in Zone I for the base-SAGD experiment quickly rose to 200 °C after 15 min of injection. In comparison, the lowest rate of temperature increase was observed in experiment #3 (100% C_4_), where it took 48 min to reach 200 °C. The temperature rise within the system was faster in base-SAGD compared to the other experiments. This is expected, as the saturation temperature of the injected vapor at the test pressure of 200 psig was highest for pure steam.

The temperatures of Zone II and Zone III eventually reached 200 °C before steam injection was terminated. However, the maximum temperature of Zone II was 173.1 °C in Experiment #3, 171.4 °C in Experiment #4, 167.5 °C in Experiment #6, 165.2 °C in Experiment #5, and 164.8 °C in Experiment #7. Similarly, the maximum temperature of Zone III was 128.4 °C in Experiment #3, 127.1 °C in Experiment #4, 122.9 °C in Experiment #6, 120.5 °C in Experiment #5, and 121.0 °C in Experiment #7. A similar trend was observed for the temperature of Zone IV, with Experiment #3 showing the highest temperature at the end of the experiment. These results suggest that a higher concentration of C_4_ in the ES-SAGD tests consistently leads to a higher temperature in the steam chamber and a faster rate of chamber expansion. Unfortunately, an issue with the ES-SAGD experiment using 100% C_3_ prevented us from injecting enough steam and solvent into the system compared to the other experiments. However, extrapolating the temperature profile toward the end of the experiment indicated that the temperature across the system was lower than in the other experiments.

The temperature of the injected fluid indicates the amount of heat provided to the system by the injector. The highest temperature of the injected fluid was observed in the base-SAGD experiment (242.5 °C), followed by Experiment #3 (234.7 °C), Experiment #6 (234.8 °C), Experiment #7 (233.7 °C), Experiment #4 (229.8 °C), and Experiment #5 (226 °C). Based on the system pressure, the injected steam quality was 100% in all tests after two pore volumes of fluid injection.

### 4.2. Cumulative Oil Production and Oil Recovery

Table 2 lists the values for irreducible water saturation, cumulative water production during oil flooding, residual oil saturation, cumulative oil production, final recovery of bitumen, water cut at the end of steam flooding, and cumulative volume of injected steam for all experiments. According to this table, irreducible water saturation varied between 5.75% and 10.12%, with an average value of 8.5%. Since the procedure for oil flooding was consistent throughout all experiments, the irreducible water value remained approximately constant. The only parameter that could affect the irreducible water saturation is the absolute permeability of the system and the structure of the pore/throat system. Table 2 confirms that the injection of the 80%C_4_–20%C_3_ mixture resulted in the highest cumulative oil production, with a final recovery factor of 99%. The residual oil saturation for this case was less than 1%. The final recovery was calculated based on the initial weight of bitumen in the sand pack and the weight of the produced bitumen during steam injection. Residual oil saturation was determined using the pore volume of the system and the difference between the initial volume of bitumen and the volume of produced bitumen at the test conditions.

By comparing the oil recovery and residual oil saturation at the end of the base-SAGD experiment with ES-SAGD #1 and ES-SAGD #2, it can be concluded that adding solvent improves the performance of base-SAGD. For instance, the injection of 7 PV of steam with propane enhanced the oil recovery from 63.03% in the base-SAGD (after 11.11 PV of steam injection) to 79.06%. Therefore, the steam-to-oil ratio significantly decreased when propane was added to the injected steam. A similar trend was observed with butane, where a lower volume of injected steam (9.4 PV) dramatically boosted oil production compared to the base-SAGD experiment. In the ES-SAGD #3 experiment, the final oil recovery reached 92.9% after injecting 9.4 PV of steam, while the base-SAGD experiment only achieved 63.03% oil recovery after injecting 11.1 PV of steam. Although the temperature of the sand pack for the base-SAGD experiment was higher (lowering the bitumen viscosity more), the addition of the solvent effectively diluted the bitumen and increased the mobility of the diluted bitumen phase. The experimental study reported by Esmaeili et al. [31] showed that adding a hydrocarbon solvent (i.e., 15 vol.% of hexane) to the bitumen enhanced two-phase bitumen/water relative permeability curves, regardless of temperature impact on relative permeability [30,33]. The mobility of the bitumen phase was improved beyond that resulting from viscosity reduction when the bitumen was diluted by a hydrocarbon solvent. The oil/water IFT measurements performed by Esmaeili et al. [31] for diluted bitumen with hexane and their comparison with the unaltered bitumen/water system [30] confirmed the lower value of IFT for diluted bitumen. The relative permeability data for diluted bitumen with propane or butane and water systems can assist in interpreting and justifying the results of oil recovery. However, the literature on these types of measurements is sparse.

The oil recovery increased by 16.0% to 30.0% compared to base-SAGD when either propane or butane was co-injected with steam, with butane performing better than propane. The higher boiling point of butane allows it to remain in the liquid phase, which reduces oil viscosity. Additionally, butane has a higher solubility compared to propane. Furthermore, due to its higher molecular weight, butane may lead to slower expansion than propane, resulting in more controlled steam distribution and improved thermal efficiency.

The key question that needs to be addressed is which composition of the propane/butane mixture results in the best oil recovery performance. The phase behavior of the bitumen/hydrocarbon/water system plays a crucial role in controlling the rate of steam condensation at the edge of the steam chamber. This is due to the partial pressures of the hydrocarbon solvent and water under the test conditions, which depend on the gas phase composition and the solubility values of propane and butane in the oil.

**Figure 4:**
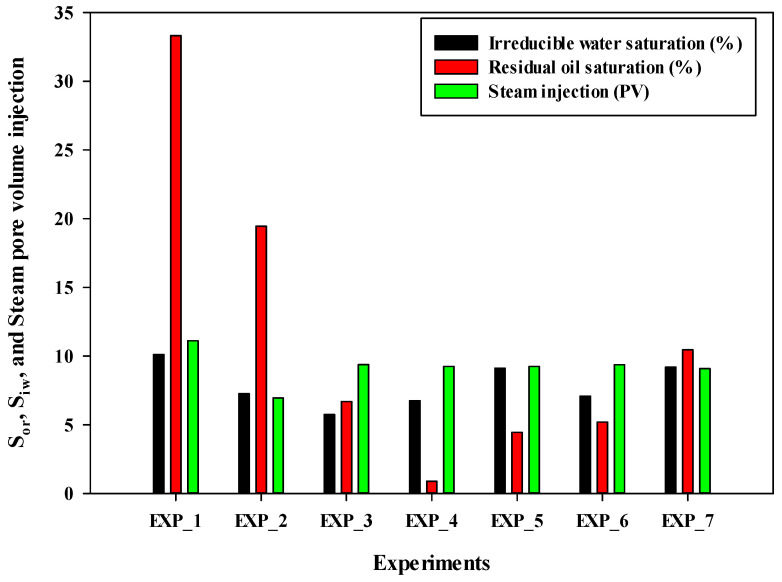
The irreducible water saturation, residual oil saturation at the end of the experiment, and total pore volume injection of steam into the sand pack in different experiments.

Figure 4 shows a bar chart illustrating the values of irreducible water saturation, residual oil saturation, and the number of pore volume injections of steam for all experiments. Experiment #4 exhibited the lowest residual oil saturation, followed by Experiment #5, Experiment #6, Experiment #3, Experiment #7, and Experiment #2. It is important to note that the volume of injected steam was nearly the same for Experiments #3 through #7. Therefore, in terms of final oil recovery and residual oil saturation, Experiment #4 demonstrated the best performance.

**Figure 5:**
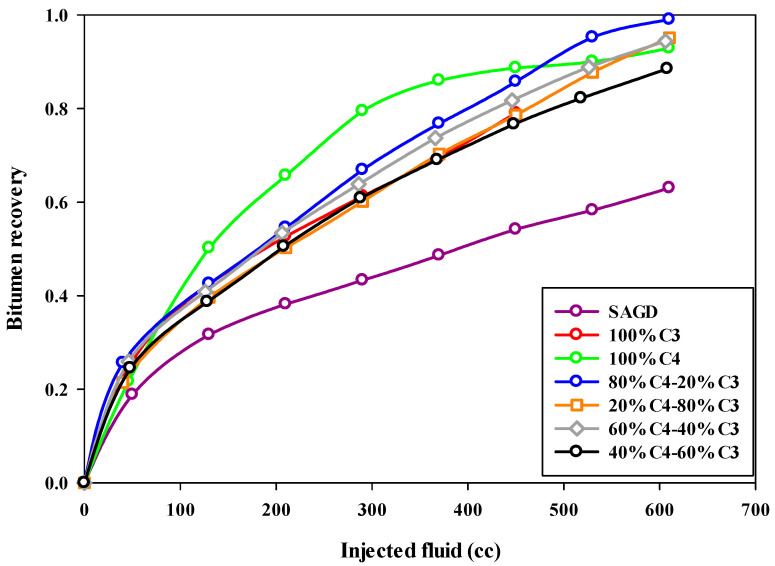
The recovery of bitumen during the SAGD or ES-SAGD process versus the volume of injected fluid at the ambient temperature in different experiments.

Both the final oil recovery and the rate of oil recovery are very important in ES-SAGD. Figure 5 compares the oil recovery trends for different experiments, showing the bitumen recovery factor versus the volume of injected fluid. The oil recovery rate was lowest in the base-SAGD experiment, reaching about half the rate of the other experiments. The cumulative oil production trend for Experiment #3, which used 100% butane along with steam injection, slightly deviated from the other ES-SAGD experiments that included propane, particularly during the middle stage of steam injection.

**Figure 6:**
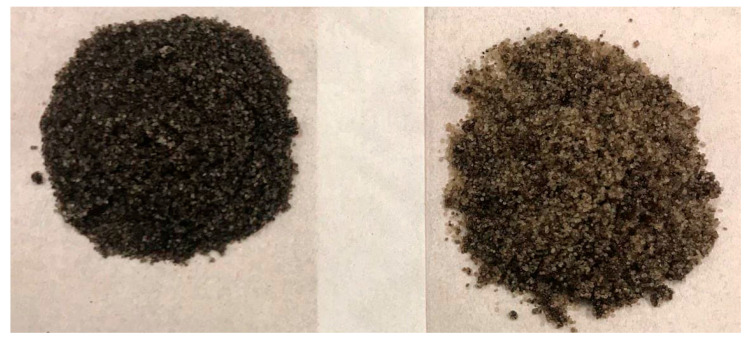
The extracted sand particle from Experiment #4 at the end of the experiment, upper part (**left side**) and lower part (**right side**) of the sand pack.

Increasing the propane fraction in the hydrocarbon solvent mixture negatively affected the oil production rate in ES-SAGD experiments. The fastest oil recovery rate was observed in Experiment #4 through Experiment #7, with Experiment #4, which had a mixture composition of 80%C_4_–20%C_3_, achieving the highest rate of oil recovery. More details are provided in Section 4.3. The injection of pure butane increased oil recovery by more than 25% compared to the base-case SAGD experiment. However, the 80%C_4_–20%C_3_ mixture resulted in an even higher ultimate bitumen recovery than pure butane. Figure 6 shows the sand particles extracted from the sand pack at the end of Experiment #4, illustrating the cleanliness of the sand particles in both the upper part (left side) and the lower part (right side) of the sand pack.

### 4.3. Steam-to-Oil Ratio and Oil Production Rate

**Figure 7:**
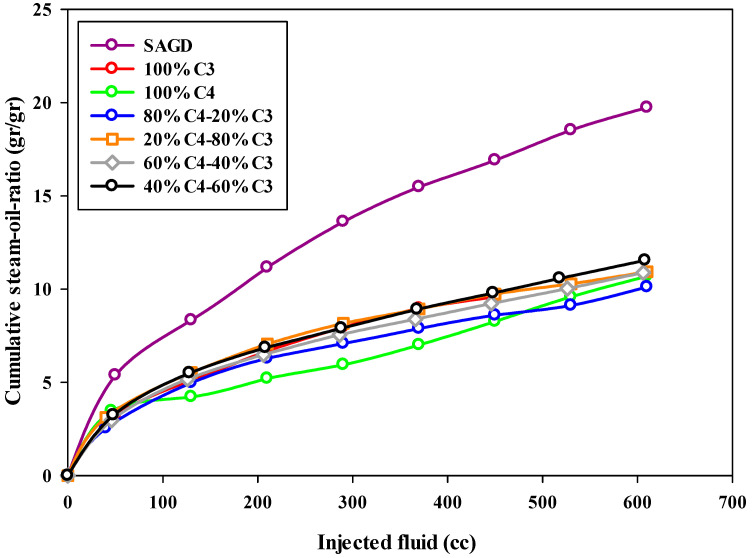
The cumulative steam-to-oil-ratio during SAGD or ES-SAGD processes versus the volume of injected fluid at the ambient temperature.

The steam-to-oil ratio (SOR) is a crucial parameter used to assess the performance of the SAGD process. This parameter discloses the efficiency of an SAGD process by determining the volume of produced oil as a function of injected steam into the reservoir. The typical value of this parameter for an economic or highly efficient SAGD process in the field is in the range of 2–10 [35]. This means that, by injecting 10 barrels of water into the reservoir, one barrel of bitumen can be produced from the reservoir. In the present study, the steam-to-oil ratio was calculated based on the mass of produced bitumen and the mass of injected water in the form of steam into the reservoir. Therefore, the reported value is in the units of gr/gr, not cc/cc. The calculated cumulative steam-to-oil ratios for all experiments at different stages are plotted versus the volume of injected fluid, either solvent or steam, in Figure 7, respectively. Since the instantaneous steam-to-oil ratio increased substantially over time, a semi-log plot was used to make the comparison easier.

According to Figure 7, the cumulative steam-to-oil ratio (SOR) for all experiments, except the base-SAGD, started at a low value (around 3.0) and increased to over 10.0 by the end of the experiment. The base-SAGD experiment exhibited the highest SOR among all tests, highlighting that adding solvent significantly improves the efficiency of the SAGD process, not only in the early stages of production but also throughout the entire experiment. The experiment using pure butane showed a low SOR (below 7.0) for the first half of the test; however, it rapidly increased in the later stages of production. This was due to the majority of the bitumen being recovered early on, leaving only a small amount of bitumen to be recovered in the remainder of the experiment. As can be found in Figure 7, the 80%C_4_–20%C_3_ mixture demonstrated an acceptable steam-to-oil ratio (SOR) throughout the entire experiment. Additionally, in all other ES-SAGD experiments using a mixture of propane and butane, the cumulative SOR was lower than that of the base-case SAGD. When comparing the cumulative SOR values from the experiments with typical SOR values for field oil reservoirs, the calculated values were found to be higher. This discrepancy can be attributed to significant heat loss during the experimental setup, especially with a small sand pack or model. Such heat loss greatly affects the performance of SAGD-type experiments, as it results in a high rate of steam condensation. In other words, the lab-scale model has a much higher surface-to-volume ratio compared to field-scale tests. Additionally, the sand pack body, made of metal, has a higher heat conductivity than rock formations. This means that much more heat can be stored in a reservoir compared to a sand pack model. We expect that the results on the field scale will be even better than those observed in the lab due to lower heat loss. While adding more insulation could help reduce heat loss to some extent, the operational conditions were consistent across all experiments, making the results comparable.

**Figure 8:**
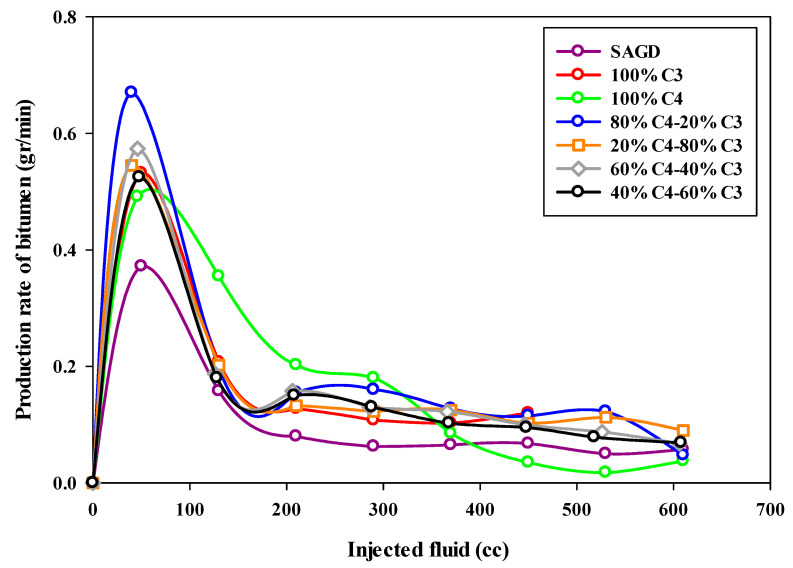
The bitumen production rate during SAGD or ES-SAGD processes versus the volume of injected fluid at the ambient temperature.

The bitumen production rate was calculated based on the total mass of produced bitumen in each sample within a particular time interval. As can be seen from Figure 8, which depicts the bitumen production rate (in grams per minute) as a function of the volume of injected fluids, the highest production rate occurred in the ES-SAGD experiment using the 80%C_4_–20%C_3_ mixture. The initial oil production rate was around 0.7 g per minute, then it declined sharply to approximately 0.2 g per minute, where it remained relatively constant for a prolonged period. In contrast, the base-SAGD experiment exhibited the lowest production rate, with the highest rate being less than 0.4 g per minute at the early stages of production, followed by a significant decline toward the end of the experiment. It was also observed that increasing the proportion of propane in the mixture led to a lower initial production rate in the mixed solvent ES-SAGD tests.

To evaluate the success and cost-effectiveness of the ES-SAGD process compared to base-SAGD, the solvent recovery should be determined. However, this is challenging in our experiments, as both C_3_ and C_4_ solvents can easily evaporate from the diluted bitumen during sample collection. Additionally, solvent recovery was not part of the scope of this study, and thus no further details are provided.

### 4.4. Final Water Cut

**Figure 9:**
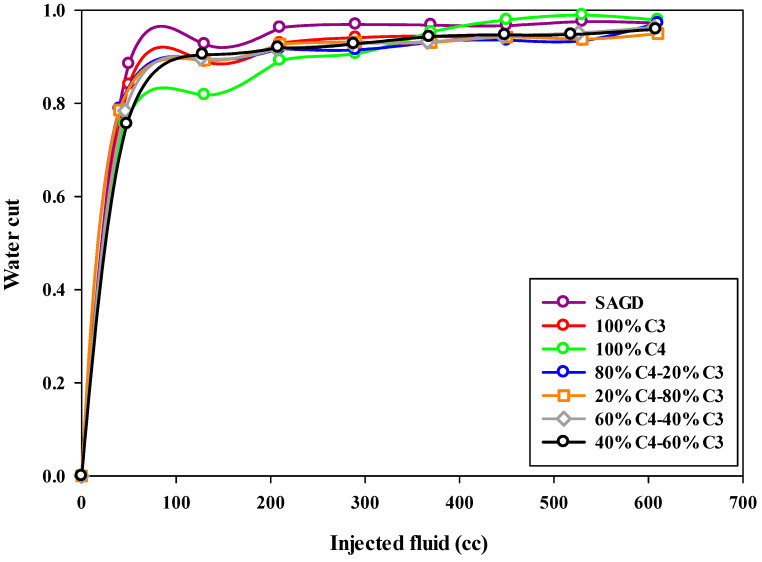
The water cut during SAGD or ES-SAGD processes versus the volume of injected fluid at the ambient temperature in different experiments.

The water cut in the produced fluid during this SAGD-type experiment was notably high. This can partly be attributed to the proximity of the production well to the injection well, which may have caused some of the steam to bypass the chamber above the injection well, leading to its production from the production well. Additionally, as previously mentioned, heat loss from the walls of the sand pack increases steam condensation, resulting in higher water production. Consequently, the water cut for both SAGD and ES-SAGD experiments was consistently high throughout the tests. Figure 9 illustrates the water cut (i.e., the water fraction in the collected effluents) for the production well across the different experiments. The highest water cut was observed in the base-SAGD experiment. When compared to the base-SAGD, the co-injection of pure butane with steam resulted in the lowest water cut during the early stages of production but the highest water cut at the later stages.

Among all the curves in Figure 9, the blue curve represents the water cut for the 80%C_4_–20%C_3_ mixture as a function of the injected fluid volume. In the early stages of production, the water cut for this experiment falls between the values observed in the other experiments. However, a notable feature of this curve is the lower water cut observed during the late stages of production. As mentioned earlier, the water cut for the base-SAGD and ES-SAGD experiments, regardless of the composition of the injected hydrocarbon solvents, remained quite high, exceeding 85% during the early stages and reaching over 92% by the end of the experiment.

### 4.5. Interfacial Tension, Solubility, and Viscosity of Solvent Additive Bitumen

The viscosity of solvent-additive bitumen has been extensively measured at different temperatures and pressures by other researchers [36,37,38,39,40], and it is confirmed that adding a little amount of solvent to bitumen significantly reduces the bitumen viscosity. The solubility of propane is lower than that of butane under the same thermodynamic conditions [36]. Although the viscosity of pure propane is lower than that of pure butane under the same pressure and temperature, the higher solubility of butane in bitumen results in lower viscosity for bitumen saturated with butane compared to propane [36]. For example, in the study by Hadadnia et al. [36] using Athabasca bitumen at 230 °C and 2 MPa, the viscosity of bitumen saturated with propane was 3.9 cP, whereas the viscosity for bitumen saturated with butane was 2.2 cP. Meanwhile, the solubility of butane was 36%, compared to only 13% for propane. Thus, diluted bitumen with butane is expected to perform better than propane in ES-SAGD.

The fluid–fluid interaction between bitumen and the solvent is another important factor influencing the performance of solvent-enhanced core flooding. Interfacial tension (IFT) is affected by factors such as molecular size, solubility, polarity, and van der Waals forces. The lower solubility of propane in bitumen compared to butane leads to a higher IFT in propane cases. Additionally, propane is more polar and has a lower molecular weight, which results in lower surface activity and less efficient IFT reduction compared to butane. For a better understanding of fluid–fluid interactions, direct measurement of IFT should be conducted under the test conditions.

### 4.6. Limitations

Laboratory-scaled core flooding tests often come with certain pros, cons, and limitations. The following points outline the key limitations of the SAGD experimental tests conducted in this research:

- One of the main limitations of the laboratory-scale core flooding tests is the significant heat loss, especially in small sand pack models. This is due to the substantial temperature differences between the ambient environment and the SAGD chamber. Furthermore, since the amount of heat injected into the system is relatively small, even minor heat loss through the piping can lead to a phase change in the system, potentially impacting the efficiency of the process. To mitigate this, working with a larger model, such as a 3D physical model, could increase the volume-to-surface ratio, thus reducing heat loss and more accurately representing field-scale conditions.
- Solvent recovery is a critical parameter in any solvent-additive SAGD project due to the high cost of solvents compared to crude oil. Although solvent recovery was not within the scope of this study, it represents a limitation of the current research. To address this issue, future experiments should collect produced samples in a pressure vessel. After cooling the system, the pressure must be gradually released, as both butane and propane can escape from the effluent. Therefore, incorporating a separator and pressure vessel in the experimental setup is necessary for accurate solvent recovery assessment. Since the effluent samples in this study were collected at ambient conditions, solvent recovery could not be properly evaluated.

## 5. Summary and Conclusions

This study examined the impact of adding light hydrocarbon solvents on the performance of the SAGD process, focusing on oil recovery, temperature profiles, and water cut. The experiment utilized Athabasca bitumen, deionized water, pure butane, propane, and four different propane/butane mixtures under identical operational conditions (outlet pressure of 200 psi). The main conclusions are as follows:

- The temperature of the steam chamber was highest in the base-SAGD experiment, where the temperature in all three zones reached that of the injected steam. In the ES-SAGD experiments, co-injecting propane reduced the chamber temperature to around 48 °C, while butane raised the temperature by 21 °C compared to propane. This difference was attributed to the partial pressure of steam in the gaseous phase, affecting condensation rates.
- Introducing light hydrocarbon solvents improved SAGD performance significantly, with oil recovery increasing by 19% to 36%. Butane was more effective than propane in enhancing oil recovery, likely due to its higher boiling point, better solubility in oil, higher thermal conductivity, and more effective viscosity reduction properties.
- The steam-to-oil ratio (SOR) was notably high in all experiments, reaching 20 g/g. This value was higher than typical field SORs due to substantial heat loss from the sand pack holder and limited space for lateral expansion of the steam chamber. The SOR was 40% lower in the ES-SAGD experiment using an 80% butane/20% propane mixture, demonstrating the benefits of solvent addition in improving SAGD performance.
- The optimal composition of the butane/propane mixture for maximum oil recovery was 80% butane and 20% propane. However, pure butane achieved the highest rate of oil production. Oil recovery exceeded 99% with the 80%C_4_–20%C_3_ mixture after injecting 9.5 PV of steam. However, more investigations need to be conducted in the future research to find the optimum concentration. It is notable that, by considering the molecular interaction between bitumen and solvents in molecular scale, the results may differ from the observation here.
- The water cut in the produced effluents was high across all experiments, consistent with the SAGD well configuration. The base-SAGD experiment had the highest water cut, while the lowest was observed at the start of the experiment with 100% butane. The water cut was lowest in the 80%C_4_–20%C_3_ mixture experiment in the later stages of production.

## Figures and Tables

**Table 1:** The properties of the experimental SAGD/ES-SAGD tests.

Properties	Base-SAGD	ES-SAGD #1	ES-SAGD #2	ES-SAGD #3	ES-SAGD #4	ES-SAGD #5	ES-SAGD #6
Mass of sand (gr)	278.5	283.6	278.6	285.3	284.1	279.6	281.3
Volume of sand (cm^3^) ^1^	105.1	107.0	105.1	107.7	107.2	105.5	106.2
Length of sand pack (cm)	31.60	31.00	31.45	30.88	30.98	31.45	31.45
Pore volume (cm^3^)	54.92	55.00	55.30	56.20	56.20	55.00	56.9
Porosity (%)	36.40	35.79	36.37	36.60	36.18	36.32	37.16
Permeability (mD)	365.4	388.3	278.6	382.7	334.4	303.8	462.7
Solvent composition	N/A	C_3_	C_4_	80%C_4_ 20% C_3_	20% C_4_ 80% C_3_	60% C_4_ 40% C_3_	40% C_4_ 60% C_3_
Steam flow rate (cm^3^/min)	2.00	1.70	1.70	1.70	1.70	1.70	1.70
Solvent flow rate (cm^3^/min)	0.00	0.30	0.30	0.30	0.30	0.30	0.30

^1^ Density of the silica sand is assumed to be 2.65 cm^3^/gr.

**Table 2:** Residual oil saturation, irreducible water saturation, final recovery, final water cut and cumulative produced oil, cumulative produced water during oil flooding, and cumulative injected water for different experiments.

Properties	Cum. Produced Water (gr) ^1^	Swir (%) ^2^	Cum. Produced Bitumen (gr) ^1^	Sor (%) ^2^	Final Recovery Factor (%) ^2^	Final Water Cut (%) ^3^	Cum. Injected Water (PV)
SAGD	49.5	10.1	31.2	33.3	63.0	97.2	11.1
ES-SAGD #1	51.1	7.3	40.4	19. 5	79.1	93.5	7.0
ES-SAGD #2	52.2	5.8	48.5	6.7	92.9	97.9	9.4
ES-SAGD #3	52.5	6.8	52.0	0.9	99.1	97.2	9.2
ES-SAGD #4	51.2	9.1	48.7	4.5	95.1	94.7	9.2
ES-SAGD #5	51.2	7.1	48.3	5.2	94.4	96.3	9.4
ES-SAGD #6	51.8	9.2	45.9	10.5	88.5	95.9	9.1

^1^ Absolute accuracy of the measurement is ±0.02 gr; ^2^ minimum accuracy of the measurement is 0.02%; ^3^ minimum accuracy of the measurement is 0.24%.

## Data Availability

The data presented in this study are available on request from the corresponding author due to confidentiality of the data and privacy concerns.

## Nomenclature

$P_{w}$	Water pressure
$S_{wir}$	Irreducible water saturation
$S_{or}$	Residual oil saturation
T	Temperature
*Greek symbols*	
$\mu_{w}$	Water viscosity
$\rho_{bit}$	Bitumen density
$\mu_{Bit}$	Bitumen viscosity
$\rho_{Wat}$	Water density
BPR	Back pressure regulator
Cum	Cumulative
GHG	Green house gases
HT	High temperature
TEOR	Thermal enhanced oil recovery
SOR	Steam-to-oil tatio
VAPEX	Vapor extraction
CSS	Cyclic steam stimulation
ES-SAGD	Expanded Solvent-SAGD
HP	High pressure
PV	Pore volume
SAGD	Steam-assisted gravity drainage
Vol.%	Volume percent
